# Small Resistance Artery Disease and ACE2 in Hypertension: A New Paradigm in the Context of COVID-19

**DOI:** 10.3389/fcvm.2020.588692

**Published:** 2020-10-30

**Authors:** María Galán, Francesc Jiménez-Altayó

**Affiliations:** ^1^Institut de Recerca del Hospital de la Santa Creu i Sant Pau, IIB Sant Pau, Barcelona, Spain; ^2^Centro de Investigación en Red de Enfermedades Cardiovasculares, Madrid, Spain; ^3^Departament de Farmacologia, de Terapèutica i de Toxicologia, Facultat de Medicina, Institut de Neurociències, Universitat Autònoma de Barcelona, Bellaterra, Spain

**Keywords:** primary arterial hypertension, angiotensin-converting enzyme 2, COVID-19, SARS-CoV2, endothelial dysfunction, renin–angiotensin–aldosterone system, oxidative and inflammatory stress, small resistance arteries

## Abstract

Cardiovascular disease causes almost one third of deaths worldwide, and more than half are related to primary arterial hypertension (PAH). The occurrence of several deleterious events, such as hyperactivation of the renin–angiotensin system (RAS), and oxidative and inflammatory stress, contributes to the development of small vessel disease in PAH. Small resistance arteries are found at various points through the arterial tree, act as the major site of vascular resistance, and actively regulate local tissue perfusion. Experimental and clinical studies demonstrate that alterations in small resistance artery properties are important features of PAH pathophysiology. Diseased small vessels in PAH show decreased lumens, thicker walls, endothelial dysfunction, and oxidative stress and inflammation. These events may lead to altered blood flow supply to tissues and organs, and can increase the risk of thrombosis. Notably, PAH is prevalent among patients diagnosed with COVID-19, in whom evidence of small vessel disease leading to cardiovascular pathology is reported. The SARS-Cov2 virus, responsible for COVID-19, achieves cell entry through an S (spike) high-affinity protein binding to the catalytic domain of the angiotensin-converting enzyme 2 (ACE2), a negative regulator of the RAS pathway. Therefore, it is crucial to examine the relationship between small resistance artery disease, ACE2, and PAH, to understand COVID-19 morbidity and mortality. The scope of the present review is to briefly summarize available knowledge on the role of small resistance artery disease and ACE2 in PAH, and critically discuss their clinical relevance in the context of cardiovascular pathology associated to COVID-19.

## Introduction

Hypertension remains the leading cause of death globally, accounting for 10.4 million deaths worldwide every year (1). Regrettably, the prevalence, morbidity, and mortality of hypertension are increasing (2). Current evidence demonstrates that alterations in small resistance artery properties are important pathophysiological features of primary arterial hypertension (PAH). Diseased small vessels in PAH show decreased lumens, thicker walls, endothelial dysfunction, and increased oxidative stress and inflammation, events that may lead to altered blood flow supply to tissues and organs, and increase the risk of thrombosis.

PAH is prevalent among patients diagnosed with coronavirus disease 2019 (COVID-19), in whom rapid disease progression has been reported. However, it is still not clear if raised blood pressure is a risk factor for increase COVID-19 lethality (3, 4). The severe acute respiratory syndrome coronavirus 2 (SARS-CoV-2), responsible for COVID-19, achieves cell entry through an S (spike) high-affinity protein binding to the catalytic domain of the angiotensin-converting enzyme 2 (ACE2), a negative regulator of the renin–angiotensin system (RAS) pathway that has been shown to have protective effects in animal models of hypertension. Given the importance of small resistance artery disease and ACE2 in PAH, it is crucial to examine their relationship with SARS-CoV-2-induced endothelial cell injury (5) to understand COVID-19 morbidity and mortality.

The objective of this review is to briefly summarize available knowledge on the role of small resistance artery disease in PAH and the contribution of the ACE2 pathway, and critically discuss their clinical relevance in the context of cardiovascular pathology associated to COVID-19.

## Small Resistance Artery Disease in Hypertension: The Contribution of the RAS Pathway

The cause of PAH is still not known in spite of it represents 90–95% of cases. A transient increase in sympathetic activity and cardiac output occurs during the early stages of the disease (6). However, the event that consistently promotes the rise in blood pressure is the increase in total peripheral resistance (7). Peripheral resistance is determined by the lumen of vessels, especially resistance vessels (i.e., microcirculation), because their small lumens (<300 μm when relaxed) extremely slow the blood flow through the arteries.

An increase in peripheral resistance occurs when lumen diameter narrows as, according to Poiseuille's law, small decreases in lumen diameter result in large increases in resistance to flow. Lumen narrowing can develop because of structural, mechanical, and functional alterations. Structural remodeling of the small arteries is a hallmark of PAH pathophysiology (8). In PAH, resistance arteries suffer from eutrophic remodeling, which increases the media/lumen ratio without a change in the media cross-sectional area, and enhanced wall stiffness (9–12). Mechanical forces on the vessel wall also contribute to hypertensive remodeling in response to altered fluid shear stress and circumferential strain (11). Additionally, functional changes can also contribute to increase peripheral resistance (12). Either an increase or a decrease in the vasoconstrictor and vasodilator influence, respectively, can promote lumen narrowing. For instance, increases in myogenic tone (13), enhanced responses to norepinephrine (14), and endothelial dysfunction (15) can contribute to increase peripheral resistance. In hypertension, the increased pulse-wave velocity resulting from large vessel stiffening induces small vessel remodeling and endothelial injury, ultimately causing microvascular damage (16). The RAS pathway controls systemic vascular resistance, by regulating blood volume and arterial pressure. When renin is released into the blood, it acts upon circulating angiotensinogen of hepatic origin to form the decapeptide angiotensin (Ang) I. Ang I is cleaved by angiotensin converting enzyme (ACE), found predominantly in the lung capillaries, which removes two amino acids from the C-terminal of Ang I to form Ang II. In 2000, two independent research groups discovered an ACE homolog, ACE2 (17, 18), which has distinct enzymatic actions and tissue distribution, and is predominantly expressed on the cell surface (19), though a soluble form exists. Importantly, classical ACE inhibitors do not affect ACE2 activity (18). ACE2 acts as a carboxypeptidase removing a single C-terminal amino acid from Ang II generating Ang-(1–7) or, less efficiently, from Ang I leading to the formation of Ang-(1–9), whereas ACE removes the C-terminal dipeptide from Ang I to form Ang II. Additionally, ACE2 cleaves a terminal residue from several other bioactive peptides including neurotensin, dynorphin A (1–13), apelin-13, and des-Arg9 bradykinin (17, 20).

Ang II is the principal effector of the RAS pathway, and causes relevant biological actions through interaction with two cell-surface G-coupled receptors: AT1R and AT2R (21, 22). Activation of AT1R is responsible for the majority of physiological and detrimental effects of Ang II, whereas AT2R activation promotes cardiovascular protection by partly opposing AT1R-induced effects (23). Ang-(1–7) is a vasodilator and mediates protective effects in the cardiovascular system through the Mas receptor, which is involved in the regulation of blood pressure and possess anti-atherosclerotic and antifibrotics effects (24, 25). Ang-(1–9) is formed from Ang I by ACE2, carboxypeptidase A, and cathepsin A, and exerts vasculoprotective actions through AT2R receptors (26, 27), though its biological actions are relatively unexplored.

Pharmacological agents targeting the RAS pathway and, specifically, the synthesis of Ang II (ACE inhibitors) or Ang II receptor signaling (Ang II receptor blockers or ARBs) are effective in reversing hypertension-induced vascular remodeling in conductive and resistance arteries (28–31). In fact, several clinical studies reported that ACE inhibitors and ARBs improve resistance vessels structure, whereas β-blockers do not (32, 33).

## Hypertension, ACE2, and SARS-Cov2 Infection

PAH is a major risk factor of mortality worldwide being its prevalence in adults high and particularly high in the elderly (34). Lately, the impact of hypertension is emphasized in the context of the novel SARS-CoV-2 infection. The severity of COVID-19 and the poor outcome of SARS-CoV-2 infected patients is commonly associated with aging, hypertension, diabetes and other cardiovascular disorders (35). Furthermore, the severity of the primary respiratory syndrome is increased in patients with pre-existing cardiovascular disease (36).

The use of RAS inhibitors is widely proven to reduce mortality in cardiovascular disease. RAS blockers are first-line drugs to treat hypertension and associated cardiovascular and renal comorbidities (37). Thus, ACE inhibitors, ARBs, and mineralocorticoid receptor antagonists are the standard therapy in hypertension and myocardial infarction (38, 39). The use of AT1R blockers and ACE inhibitors is encouraged in hypertensive patients because these drugs are vasoprotective, and their associated increase in ACE2 expression (see the paragraph below) protects against hypertension (3, 4, 40). Discontinuation of this therapy leads to deterioration of cardiac function and heart failure with a possible increase in mortality within a short period of time (41).

Solid evidence from human and rodent studies suggests that inhibition of RAS by AT1R blockers leads to upregulation of ACE2 (42, 43). Nevertheless, evidence of ACE inhibitors affecting the expression of ACE2 is more limited (3, 44). Recently, the hypothesis that ACE inhibitors could act as a potential risk factor for fatal COVID-19 by up-regulating ACE2 was proposed (45, 46). However, there is enough evidence that allows stating also the opposite hypothesis. Indeed, there is currently no clinical data evidencing a direct link between ACE2 activity and SARS-CoV-2 associated mortality or between RAS inhibitors intake and impaired outcome in COVID-19 (4, 47). Recently, Sama et al. (2020) reported that neither ACE inhibitors, ARBs, nor mineralocorticoid receptor antagonists were associated with ACE2 concentrations in plasma in a wide cohort of patients with heart failure, albeit a group at high risk for COVID-19 (48). Furthermore, ACE2 is a target for several coronaviruses and influenza viruses, and its expression and signaling pathway is severely affected by pneumonia virus infection (49–51). The decrease of surface ACE2 levels leads to increased Ang II local levels, an effect that probably contributes to the significant mortality rates resulting from SARS-induced acute lung injury and fibrosis (49, 52). Therefore, cardiovascular protection induced by ACE2-induced degradation of Ang II and increase of Ang-(1–7) might be compromised (53–56), leading to RAS overstimulation (57).

ACE2 is involved in infection and pathology induced by SARS-CoV and the new SARS-CoV-2 which is causing COVID-19 pandemic, through its unexpected function as the cell-surface receptor for the virus facilitating viral RNA entry in the lungs (58). Since the SARS-CoV outbreak in 2002, extensive structural analyses has revealed key atomic-level interactions between the SARS-CoV spike protein receptor-binding domain and its host receptor ACE2, which regulate both the cross-species and human-to-human transmissions of SARS-CoV. The spike glycoprotein (S protein) of SARS-CoV on the virion surface mediates receptor recognition and membrane fusion. During viral infection, the trimeric S protein is cleaved into S1 and S2 subunits, and S1 subunits are released (59, 60). S1 directly binds to the extracellular peptidase domain of ACE2 through the receptor-binding domain, which in turn is recognized by the peptidase domain of ACE2 (61, 62), whereas S2 is responsible for membrane fusion. An N-terminal peptidase domain and a C-terminal collectrin-like domain, which ends with a single transmembrane helix and a ~40-residue intracellular segment, form full-length ACE2. The sequence of the 2019-nCoV spike protein (S protein), including its receptor-binding motif that directly contacts ACE2, is similar to that of SARS-CoV. Moreover, several critical residues in 2019-nCoV receptor-binding motif (particularly Gln493) provide favorable interactions with human ACE2, consistent with 2019-nCoV's capacity for human cell infection (63, 64). In principle, the virus has limited potential to escape soluble ACE2 mediated neutralization without simultaneously decreasing affinity for native ACE2 receptors, thereby attenuating virulence. Soluble ACE2 has proven safe in healthy human subjects and 45 patients with lung disease (65, 66), and recombinant soluble ACE2 is being tested in a clinical trial for COVID-19 in Guangdong province, China (Clinicaltrials.gov #NCT04287686).

## Small Resistance Artery Disease and ACE2 in COVID-19-Related Vascular Pathology

Advanced age, hypertension, diabetes mellitus and obesity, are all among the risk factors associated with a poor outcome in COVID-19. These cardiovascular disease risk factors show a common link: they are associated with pre-established vascular dysfunction. This evidence rises the hypothesis an environment of deteriorated vascular cell function is more prone to SARS-CoV-2 pathogenesis. Thus, SARS-CoV-2-infected microvascular endothelial cells (ECs) may exacerbate endothelial dysfunction (5).

### Evidence of Hypertension-Like Small Resistance Artery Disease in COVID-19

The endothelium is a crucial regulator of vascular tone by releasing vasoconstrictors and vasodilators that contribute to vessel homeostasis. Its function is impaired in hypertensive patients, with the presence of reduced vasodilation (i.e., endothelial dysfunction), increased vascular tone, inflammation and thrombosis. In addition, ECs are linked to adjacent cells to form cellular barriers between the blood and tissues that restricts the movement of water, proteins, certain chemicals, and blood cells (67). Evidence indicates that SARS-CoV-2 is able to infect ECs from lung capillaries leading to the development of acute respiratory distress syndrome (68). Pre-existing endothelial dysfunction due to aging is aggravated with the infection of vascular cells by SARS-CoV-2 (5). Thus, patients with severe COVID-19 show vascular leakage and pulmonary edema, because of EC dysfunction, lysis, and death (69). Notably, in patients with COVID-19, EC infection occurs in tissues distal from the primary infection site, leading to multi-organ failure (68). These outcomes could be the result of the disruption of the pulmonary EC barrier under the hypothesis that endothelium is a crucial target of SARS-CoV-2, which permits the virus to spread to distant target organs and may explain its systemic manifestations (70). A further consequence of endothelial damage in COVID-19 is the excessive activation of coagulation pathways (69), a common feature in hypertensive patients.

Endothelial dysfunction in hypertension is partly due to the presence of inflammatory and oxidative stress. Increased oxidative and inflammatory stress induced by activated immune cells, inflammatory cell infiltration, and vasoactive molecules promoting vasodilation, all contribute to EC de-structuring and dysfunction, which facilitate the amplification of the inflammatory response. The pulmonary microvascular ECs with inflammatory phenotype are more prone to vascular permeability, which facilitates neutrophil extravasation, and initiate arteriolar vasoconstriction (71). In addition, viral pneumonia activates innate immune response by increasing the release of inflammatory mediators, which can induce systemic inflammatory response syndrome. Although respiratory failure because of respiratory distress syndrome is the primary cause of mortality (72), many patients with COVID-19 exhibit a secondary exaggerated inflammatory response called “cytokine storm,” a hyperinflammatory syndrome characterized by a fulminant and fatal hypercytokinaemia with multiorgan failure (73). The cytokine storm leads to pulmonary parenchymal inflammation and edema that interfere with alveolar gas exchange and results in hypoxemia. Hypoxia and carbon dioxide retention cause the reflex spasm of pulmonary blood vessels ending in pulmonary hypertension. The high levels of cytokines in these patients under an environment of EC dysfunction may amplify the cascade of events leading to multi-organ failure and death. In fact, immune dysregulation observed in severe course of COVID-19 is similar to immune dysregulation in hypertension (74). CD4^+^ T-cells, and in particular CD8^+^ T-cells, are abnormally regulated in hypertension, showing greater production of pro-inflammatory cytokines (75). Moreover, hypertension is associated with a characteristic immunosenescent profile in CD8^+^ cells, which is prone to overproduction of cytokines, while are less efficient in antiviral defense (75, 76).

### Potential Impact of SARS-CoV-2–Induced “Cytokine Storm” on Small Resistance Artery Properties

Recently, the pro-inflammatory cytokine and chemokine profile associated with COVID-19 disease severity, driving a more severe and fatal clinical course, has been unveiled in two Wuhan (China) populations. Several clinical studies have shown a notably increase in circulating levels of different interleukins, C-reactive protein, granulocyte-colony stimulating factor, interferon-γ inducible protein 10, monocyte chemoattractant protein 1, macrophage inflammatory protein 1-α, and tumor necrosis factor-α (77, 78). Consistently, interleukin-1β, interleukin-2, and interleukin-6 were identified decades ago as predictors of outcome in severe adult respiratory distress syndrome (79). Furthermore, tumor necrosis factor-α and interleukin-1β activate ECs to initiate coagulation pathways by expressing P-selectin, von Willebrand factor and fibrinogen (80), an effect that might partly explain the hypercoagulability observed in COVID-19 patients.

It is noticeable that several of these cytokines have been previously associated with small vessel disease. Receptors for tumor necrosis factor-α and interleukin-1β are expressed in both ECs and smooth muscle cells (SMCs) (81, 82). Long-term exposure to both cytokines can either reduce or increase vasoconstrictor responses, an effect similar to that induced by exposure to interleukin-6 (83). In rat resistance arteries, either subchronic “*in vivo*” (84) or “*in vitro*” (85) exposure to interleukin-1β and interleukin-6 reduce acetylcholine-mediated relaxation, which is a hallmark of endothelial dysfunction commonly related to cardiovascular disease (15). The cytokine-induced endothelial dysfunction is associated with an increase in superoxide anion production that reduces nitric oxide bioavailability (85). Importantly, superoxide anion causes higher vasoconstriction in rat pulmonary vs. systemic arteries, suggesting that the pulmonary artery bed may be more prone to cytokine-induced vascular dysfunction (86). Overall, the effects of cytokines on vascular reactivity are complex, and they are vascular bed and exposure time dependent, with short times inducing a direct effect and longer times involving the contribution of crucial secondary mediators such as nitric oxide, prostanoids, and endothelin (83).

Because of available data show elevated plasma levels of certain inflammatory cytokines in some COVID-19 subpopulations, a cytokine storm-targeted rescue therapy for patients with COVID-19 infection who exhibit rapid disease progression has been proposed (87). Nevertheless, an anti-cytokine approach has not yet been proven safe and effective. Several ongoing clinical trials are investigating the use of tocilizumab, an interleukin-6 receptor inhibitor, as a potential treatment for COVID-19 (88), and a small (21 patients with severe or critical COVID-19) clinical trial in China (ID: ChiCTR2000029765) has shown encouraging results. Other studies have observed that those patients with COVID-19 and hyperinflammation, could benefit from corticosteroids treatment, which induces immunosuppression that could improve mortality (73), whereas in those patients not showing hyperinflammation corticosteroids might cause further lung injury (89).

### Small Resistance Artery Disease and ACE2 in Vascular Pathology

ACE2, which is highly expressed in SMCs and ECs, regulates cellular responses to inflammation (90). SARS-CoV-2 binds to ACE2 reducing its activity (49–51, 91), which leads to RAS overstimulation (54). ACE2 deficiency exacerbates vascular injury (92), whereas reduced ACE2 activity can increase neutrophil infiltration and induce lung inflammation (93). In addition, ACE2 protects from experimental acute lung injury (94). Stimulation of the ACE2/Ang-(1–7)/Mas axis reduces SMC proliferation (95), migration (96), endothelial dysfunction (97), and thrombosis (98). Furthermore, previous studies have demonstrated that selective ATR2 activation suppresses the action of inflammatory cytokines both “*in vitro*” and “*in vivo*” (99–102). However, only few works have reported the association between ACE2 and ATR2 (103, 104). Activation of ATR2 enhances ACE2 expression and activity in human ECs contributing to the anti-inflammatory effects of ATR2-mediated signaling (105). Furthermore, ACE2-induced activation of ATR2 by Ang-(1–7) after ATR1 blockade is associated with improvement of vascular remodeling (53).

## Conclusions and Upcoming Perspectives

**Figure 1:**
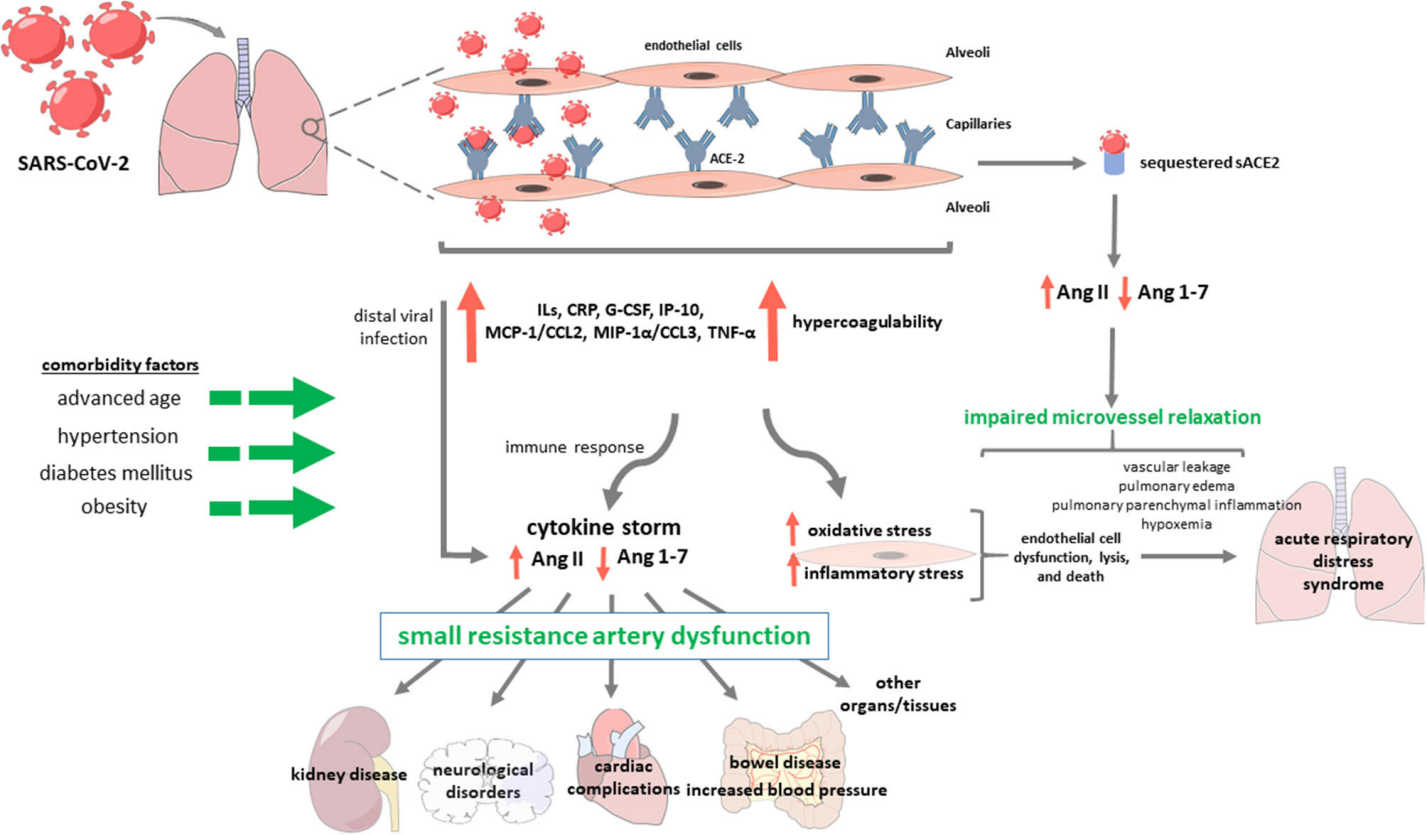
Potential role of SARS-CoV-2, responsible for COVID-19, in small resistance artery dysfunction and organ/tissue injury. The SARS-Cov2 virus infects endothelial cells from lung capillaries because it achieves cell entry through an S (spike) high-affinity protein binding to the catalytic domain of angiotensin-converting enzyme 2 (ACE2). The virus causes endothelial damage by increasing pro-inflammatory cytokines and chemokines expression and excessive activation of coagulation pathways. Furthermore, the interaction of SARS-CoV-2 with ACE2 compromises ACE2-induced degradation of angiotensin (Ang) II and reduces Ang-(1–7) levels, leading to renin–angiotensin system overstimulation. Altogether, these events may contribute to endothelial cell dysfunction and death, which can induce vascular leakage, pulmonary edema and parenchymal inflammation, hipoxemia and, ultimately, acute respiratory distress syndrome. Notably, in patients with COVID-19, peripheral manifestations of endothelial dysfunction occur in tissues distal from the primary infection site, probably because of the disruption of the pulmonary endothelial cell barrier that permits the virus to spread to distant target organs, and/or due to the secondary exaggerated inflammatory response (cytokine storm). This endothelial damage would cause small resistance artery dysfunction and alter blood flow supply to tissues and organs, increasing the risk of thrombosis and multi-organ failure. The presence of cardiovascular disease risk factors such as advanced age, hypertension, diabetes mellitus and obesity, which are associated with pre-existing endothelial dysfunction, may worsen the above-mentioned pathological mechanisms leading to poor outcome in COVID-19. IL, interleukins; CRP, C-reactive protein; G-CSF, granulocyte-colony stimulating factor; IP-10, interferon-γ inducible protein 10; MCP-1/CCL2, monocyte chemoattractant protein 1; MIP-1α/CCL3, macrophage inflammatory protein 1-α; TNF- α, tumor necrosis factor-α.

Few works have been focused on describing the anomalies and potential underlying mechanisms of small resistance artery disease in COVID-19 (Figure 1). The dysfunction of these vessels, which resembles those from hypertensive patients, seems crucial for the development of severe COVID-19, and for the long-term target organ damage observed during the follow-up of these patients.

The present review highlights the beneficial roles of ACE2 signaling in small arteries through the control of the RAS pathway. The reduction of circulating levels of Ang II and the anti-inflammatory actions of Ang-(1–7) and ATR2 signaling are the main mechanisms involved in ACE2-induced vasculoprotection. Nevertheless, the role of ACE2 in the anti-inflammatory actions of ATR2 has not been studied “*in vivo*,” which warrants further investigation. Available evidence suggests that the use of pharmacological interventions to increase NO bioavailability, enhance Ang-(1–7) and ATR2 signaling, and improve ACE2 activity might have a positive impact on small resistance artery disease in either hypertension or COVID-19 (5, 106, 107).

## Author Contributions

FJ-A and MG conceived and drafted the manuscript. Both authors revised the manuscript for important intellectual content and gave their final approval of the submitted version.

## Conflict of Interest

The authors declare that the research was conducted in the absence of any commercial or financial relationships that could be construed as a potential conflict of interest.
